# Dual detection high-speed capillary electrophoresis for simultaneous serum protein analysis and immunoassays

**DOI:** 10.1038/s41598-022-05956-8

**Published:** 2022-02-04

**Authors:** Prabhavie M. Opallage, Miyuru De Silva, Robert C. Dunn

**Affiliations:** Ralph N. Adams Institute for Bioanalytical Chemistry, Department of Chemistry, University of Kansas, 2030 Becker Drive, Lawrence, KS 66047 USA

**Keywords:** Bioanalytical chemistry, Microfluidics

## Abstract

Serum protein electrophoresis (SPE) separates serum proteins into bands whose shape and amplitude can alert clinicians to a range of disorders. This is followed by more specific immunoassays to quantify important antigens and confirm a diagnosis. Here we develop a high-speed capillary electrophoresis (HSCE) platform capable of simultaneous SPE and immunoassay measurements. A single laser excitation source is focused into the detection zone of the capillary to measure both refractive index (SPE) and fluorescence signals (immunoassays). The refractive index signal measures characteristic SPE profiles for human serum separated in 100 mM boric acid (pH 10), 100 mM arginine (pH 11), and 20 mM CHES (pH 10). For the immunoassay, the fluorescence electropherograms reveal that CHES provides the optimal buffer for measuring the immunocomplex and separating it from the free antigen. Immunoassays in CHES yield a LOD of 23 nM and a LOQ of 70 nM for the detection of fluorescein. The high pH reduces protein adsorption but reduces antibody affinity. Preliminary studies carried out in 50 mM barbital at pH 8 show improved stability of the immunocomplex and better separation for immunoassay quantification. Further optimization will open new capabilities for measuring orthogonal diagnostic signals in seconds with HSCE.

## Introduction

Serum protein electrophoresis (SPE) is an important tool used clinically to separate serum proteins into five major bands—albumin, alpha-1, alpha-2, beta, and gamma^1–4^. Electrophoretic separation of serum proteins was first demonstrated in the early 1900s by Tiselius using a U-shaped electrophoretic cell^5^. This combination of moving boundary electrophoresis coupled with optical detection, marked the first separation of serum proteins into distinct protein bands^5^. Since these early studies, electrophoretic separations using cellulose acetate or more commonly agarose gels rapidly evolved and became the gold standard for SPE measurements^6^. Unlike the moving boundary experiments, the gels provided a stable matrix to immobilize and stain the protein bands following separation, enabling quantification using densitometry. More recently, capillary zone electrophoresis (CZE) has emerged as an attractive alternative for SPE given its advantages in throughput and automation^7–9^.

Protein separation with CZE first began appearing in the mid-1980s, with the first commercial instrument dedicated to clinical SPE measurements introduced in the late 1990s^10,11^. SPE with CZE is normally performed in buffers with high pH and ionic strengths to negatively charge the proteins and reduce their interactions with the capillary walls^11^. Protein adsorption to the capillary wall is a common problem that can substantially degrade separation performance^12^. Other commonly used approaches to reduce protein adsorption involve dynamic coatings added into the background electrolyte (BGE) or chemical modifications of the capillary wall^13–17^. Once separated, proteins are detected directly using UV absorption. Since labeling steps are not required, this approach is convenient, but also restricts the choice of buffers to those that are transparent in the UV spectral region.

Beyond the advantages of throughput and automation, CZE also has better separation efficiency and repeatability compared with agarose gel SPE^18,19^. The C_3_ and transferrin peaks in the beta region are better resolved in CZE and there is improved quantification of the pre albumin portion of the SPE^20^. The gamma and beta bands migrate first in CZE causing them to narrow while the alpha and albumin bands are broader compared with agarose gel electrophoresis^3^. Sensitivity is also slightly better with CZE compared to agarose gel, while specificity seems somewhat lower although the differences are small^19^. The protein staining required for agarose gel SPE can vary with protein concentration and lead to unwanted background staining of the support media^21^. There can also be challenges associated with non-uniform and non-linear dye binding, which can complicate and degrade quantification with the densitometry measurements in gel SPE^6^. CZE, on the other hand, predominantly uses direct detection of proteins using UV absorbance at 214 nm. This eliminates the time and complications arising from protein staining but can lower specificity since all species absorbing in the UV will contribute to the SPE profile. The presence of antibiotics, for instance, has been shown to lead to unwanted contributions to the SPE signal^22^.

SPE profiles, measured with gel or CZE, can alert clinicians to a spectrum of disorders since trauma or disease often upsets the balance of serum proteins^23^. Decreased levels of albumin, for example, can be indicative of a hereditary deficiency (hypoalbuminemia) or disease states like nephrotic syndrome^24^. Likewise, primary amyloidosis and immune deficiencies reduce the levels observed in the gamma region^2^. The most prevalent use of SPE, however, is in the detection and diagnosis of monoclonal gammopathies, such as multiple myeloma^1,2,25^. These disorders arise from the proliferation of a single B-cell clone, leading to the excess production of a monoclonal immunoglobulin^1,26^. Since immunoglobins migrate largely in the gamma band, monoclonal gammopathies result in a characteristic spike in the gamma region of the SPE^1,2^. An unusual profile in the SPE triggers a series of more specific immunoassays to quantify important antigens and confirm a diagnosis^2^. Clinically, enzyme-linked immunosorbent assays (ELISA) remain the most popular format for immunoassays. However, a large body of work has shown that CZE is also suitable for immunoassay measurements using a myriad of approaches^27–29^. This opens intriguing possibilities for combing both SPE and immunoassays measurements using one CZE platform.

Antigen–antibody complexes initially at equilibrium can be separated from unbound species in CZE, as long as the electrophoretic mobilities of the species are sufficiently different and the separation time is competitive with the equilibrium kinetics^30^. Affinity-based CZE techniques have been thoroughly discussed in the literature and identified as affinity probe capillary electrophoresis (APCE) and more recently as non-equilibrium capillary electrophoresis in equilibrium mixtures (NECEEM)^30–35^. NECEEM, in particular, has advantages since it does not require an equilibrium to be established inside the capillary^36^. Unlike other approaches, it does not require antibody or antigen in the BGE and separations are carried out on pre-equilibrated samples using conventional injection and separartion conditions, making simultaneous serum protein analyisis and immunoassays possible. CZE analysis of pre-equilibrated solutions were first used to analyze human growth hormone, where bound and unbound species were successfully separated and quantified using UV detection^37,38^. The integration of fluorescence labeling methods increased specificity and substantially improved detection limits, expanding the reach of the technique^27^. Finally, methods using either labeled antigen or antibody have been developed along with strategies for multiplexed detection^33,39,40^.

APCE, NECEEM, and related CZE based immunoassays are homogenous assays, which has advantages over more traditional techniques like ELISA, where surface immobilization degrades antibody performance. CZE methods also require very little sample and have enhanced specificity due to the separation step. The latter also improves the workflow since CZE based immunoassays do not require the multiple fluid exchanges and rinsing steps required in traditional immunoassays. Finally, in addition to quantifying analyte concentration, CZE measurements can yield fundamental parameters like K_d_ and rate constants^41,42^. This has led to a number of CZE based assays for drug binding^32,43–49^, hormone analysis^50–53^, virion analysis^54–57^, along with other applications^58^.

The total analysis time is an important consideration when using APCE or NECEEM with preincubated samples, since the antigen–antibody complex continually dissociates during the non-equilibrium separation^58^. Shortening the length-to-detector, elevating the separation field, and increasing the electroosmotic flow (EOF) are all strategies that have been used to decrease analysis times^59^. These approaches have been successful in performing separations in under a minute, which is sufficient for preventing significant dissociation of most immunocomplexes^27^.

Combining SPE and immunoassay measurements on one separation platform can consolidate assays and streamline workflow. For instance, once a M-spike is observed and multiple myeloma is suspected, a series of immunosubtraction and immunoassay measurements are normally carried out to isotype the monoclonal antibody, confirm the diagnosis, and stratify risk. Isotyping the antibody using immunosubtraction requires a series of SPE measurements in CZE. Each SPE run provides an opportunity to measure free light chains and other important biomarkers such as β2 microglobulin simultaneously. This work discusses the development of a high-speed capillary electrophoresis (HSCE) platform capable of simultaneous SPE and immunoassay measurements.

## Results and discussion

**Figure 1:**
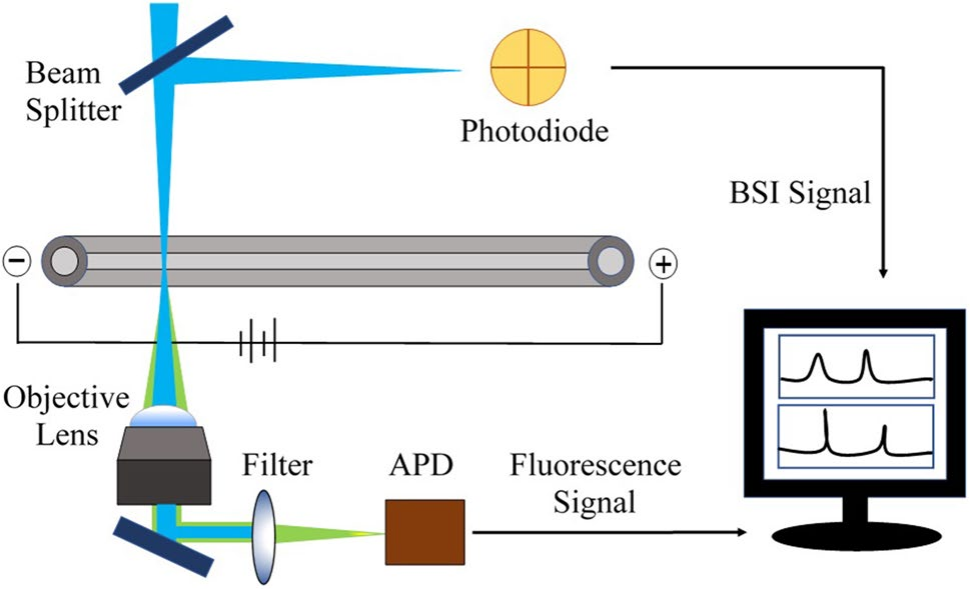
Schematic of the dual detection HSCE platform using a single laser excitation source to generate the BSI and fluorescence signals. Excitation light is focused into the detection zone of the capillary and reflected back to form the BSI fringe pattern. A beam splitter reflects the fringe pattern towards a split photodiode detector aligned on two of the fringes. The differential output is amplified and recorded as the BSI signal. Fluorescence excited by the same excitation source is collected from below the capillary with an objective, filtered, and detected with an APD. Both signals are recorded simultaneously.

The dual detection HSCE is shown schematically in Fig. 1. The laser source is focused into the detection zone of the separation capillary and the back-scattered radiation is detected on a segmented photodiode detector. For the experiments described here, the 488 nm line of an argon-krypton laser is used for excitation. As shown previously, the interference pattern in the backscattered radiation responds to changes in refractive index (RI) as analytes pass through the detection zone^60,61^. Shifts in the interference pattern are measured as intensity changes on the split photodiode. The differential signal from the two elements is amplified and recorded as the BSI signal. Since RI is a universal detection signal, proteins and other species in the serum sample will all contribute to changes in the BSI signal.

**Figure 2:**
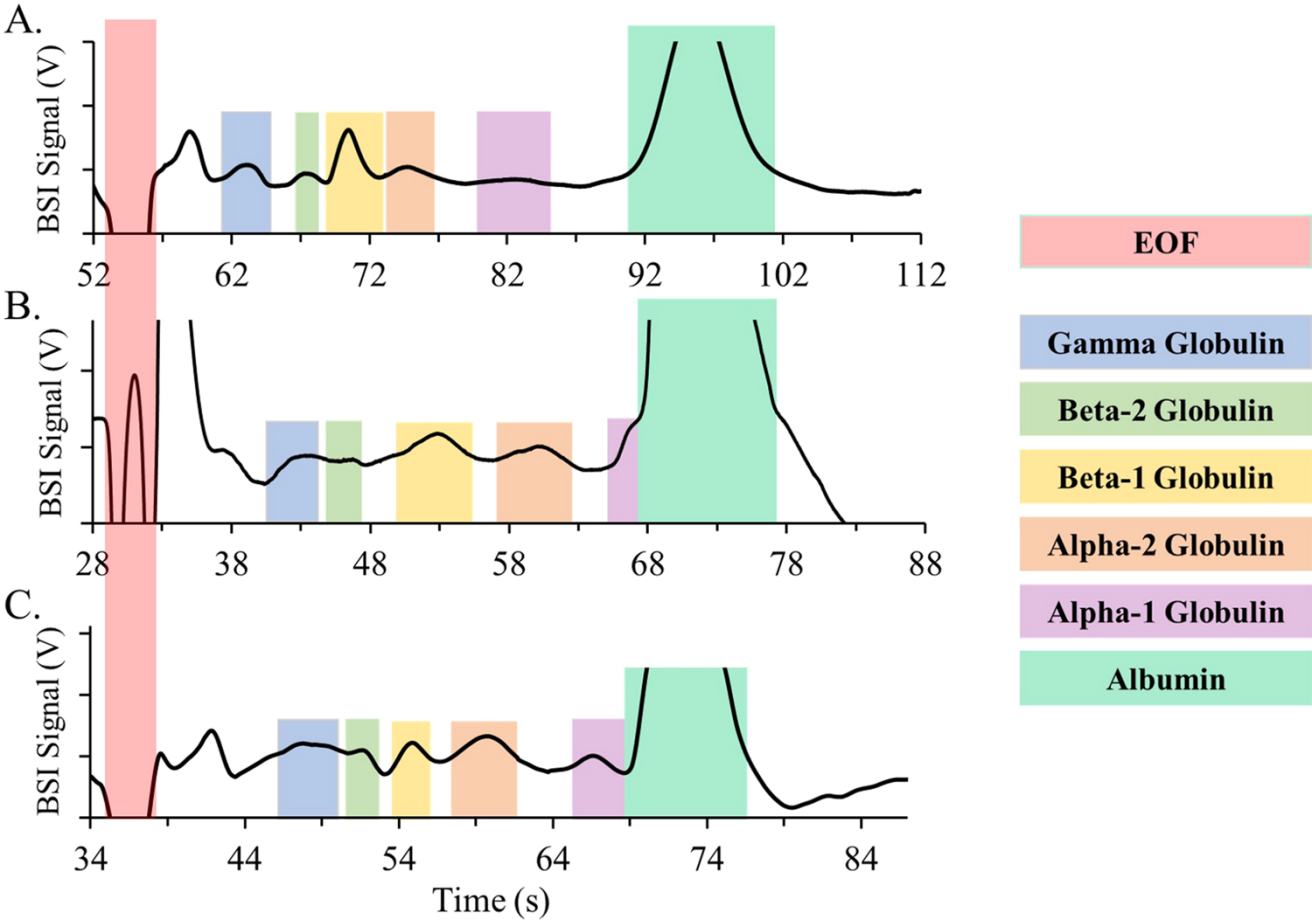
Serum protein electropherograms (SPE) measured in various buffers using HSCE combined with BSI detection. Human serum samples separated in (**A**) 100 mM boric acid at pH 10 (400 V/cm), (**B**) 100 mM arginine at pH 11 (500 V/cm), and (**C**) 20 mM CHES at pH 10 (500 V/cm). Each SPE contains six major protein bands as identified and were separated in normal polarity, where the proteins migrate following the EOF, which is the large spike identified at short times in each electropherogram. The large peak due to albumin saturates the signal in each electropherogram.

Figure 2 shows representative SPE profiles measured using BSI in the HSCE platform. Three different buffer systems are compared, all with a pH above typical protein pI values to negatively charge the proteins and reduce interactions with the capillary wall^62,63^. In normal polarity mode, the negatively charged proteins electrophoretically migrate towards the anode but are swept towards the cathode and past the detection zone by the electro-osmotic flow (EOF). The migration time, therefore, reflects the difference between these two processes^20^.

Figure 2A shows the SPE electropherogram measured in 100 mM boric acid at pH 10. Given its transparency in the UV spectral region, this is traditionally the most utilized buffer system for SPE measurements with CZE^20^. The serum sample (10 mg/mL) was hydrodynamically injected (65 kPa, 150 ms) into the HSCE capillary and separated at a field strength of 400 V/cm. As shown in Fig. 2A, all five major protein bands are identified in the electropherogram. Even with the ultra-thin wall capillary that efficiently dissipates heat, we observe the onset of Joule heating at field strengths above 400 V/cm using 100 mM boric acid. Since high field strengths are required for the rapid separations desirable for immunoassays, low conductivity buffer systems were explored.

Figure 2B, C show SPE electropherograms measured in BGE of 100 mM arginine at pH 11 and 20 mM N-cyclohexyl-2-aminoethanesulfonic acid (CHES) at pH 10. The zwitterionic nature of these buffers reduces the conductivity of the BGE, enabling higher separation fields (500 V/cm) without Joule heating. As shown in Fig. 2, the migration time for the last SPE peak (albumin) in both optimized buffers is reduced from 96 s in boric acid to under 79 s in the zwitterionic BGEs at the higher field strength. All five major protein bands are resolved in both arginine and CHES and, importantly, the gamma globulin band in which immunoglobulins migrate is shifted farther from the EOF band compared with comparable measurements in boric acid. Since all five major protein bands are resolved in the electropherograms shown in Fig. 2, all three optimized buffer systems were deemed suitable for SPE with HSCE.

To implement simultaneous immunoassay measurements, a second detection channel based on fluorescence is introduced. As shown schematically in Fig. 1, the same excitation beam used to generate the BSI signal excites fluorescence in the detection zone of the capillary. The fluorescence is collected from below using an objective, filtered to remove residual excitation light, and imaged onto a single-photon avalanche diode (SPAD) detector. Since both the BSI and fluorescence signals are derived from the same excitation source, both signals arise from the same detection volume in the capillary and provide synchronized and orthogonal signals of sample properties.

To validate and optimize simultaneous SPE and immunoassays measurements, a monoclonal antibody specific for the fluorescent dye fluorescein was utilized. As discussed, there have been many methods developed for combining immunoassays with separations^27–29^. To maintain the SPE signal, however, approaches like NECEEM that begin with pre-equilibrated samples are the most straightforward to implement^30–35^. The three buffer systems optimized for SPE in Fig. 2, therefore, were tested for their ability to support successful immunoassays. To be successful, the bound and unbound species must be sufficiently separated and the total analysis time should be competitive with the immunocomplex dissociation kinetics^31^. To test the suitability of the BGEs, initial experiments used serum samples (7 mg/mL) pre-incubated for 30 min with 38 nM fluorescein and excess monoclonal antibody (334 nM).

**Figure 3:**
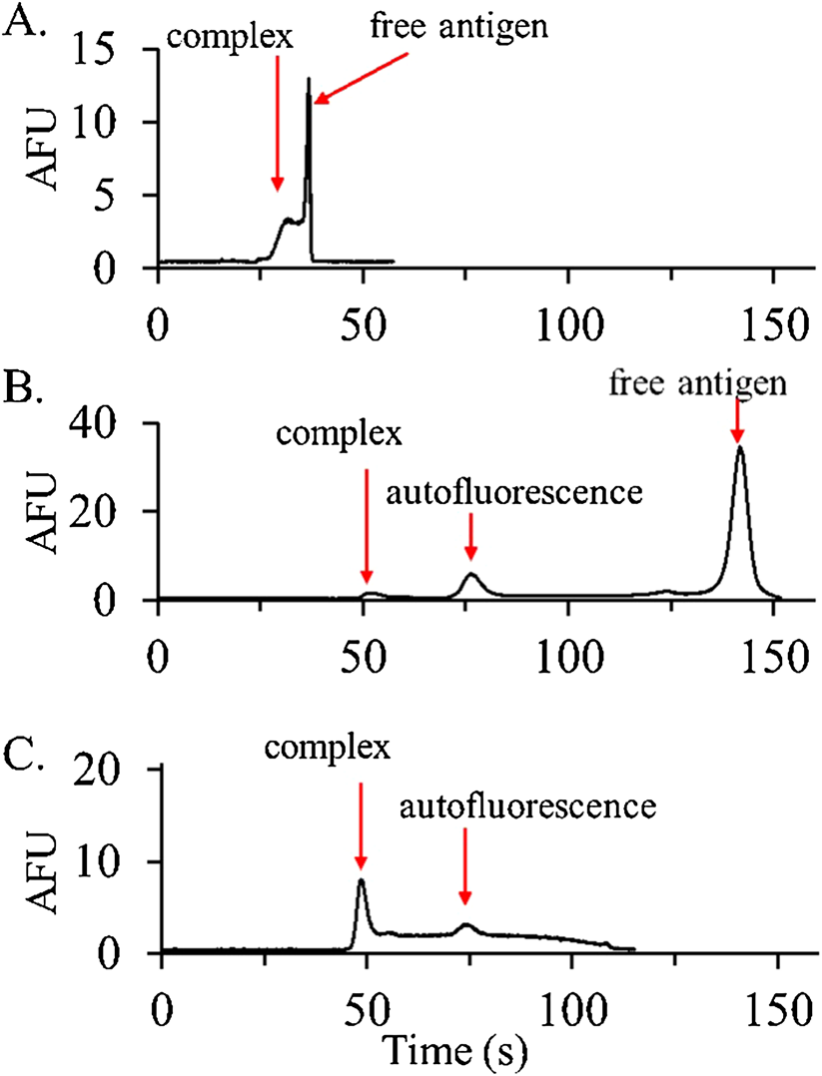
HSCE fluorescence electropherograms for serum samples incubated with 334 nM anti-fluorescein antibody and 38 nM fluorescein antigen. The mixtures were separated in background electrolytes of (**A**) 100 mM arginine at pH 11 (500 V/cm), (**B**) 100 mM boric acid at pH 10 (400 V/cm), and (**C**) 20 mM CHES at pH 10 (500 V/cm). Poor separation and low population of the immunocomplex make boric acid and arginine, respectively, unsuitable for this immunoassay. The electropherogram in CHES, on the other hand, shows a strong immunocomplex peak, making it suitable for the immunoassay.

Figure 3 shows fluorescence electropherograms for pre-incubated serum samples in the three buffer systems previously optimized for SPE – 100 mM arginine (Fig. 3A), 100 mM boric acid (Fig. 3B), and 20 mM CHES (Fig. 3C). The electropherogram in Fig. 3A shows that the immunocomplex and free antigen are poorly separated in the 100 mM arginine at pH 11, making this BGE unsuitable.

The electropherogram in Fig. 3B, on the other hand, shows that the immunocomplex and free antigen are fully separated in boric acid at pH 10, but little complex remains following the separation. This suggests that significant dissociation of the immunocomplex has taken place during the separation, making this BGE problematic. Finally, the electropherogram in Fig. 3C measured in CHES at pH 10, shows a strong peak for the immunocomplex making this BGE appropriate for immunoassays. The broad background trailing the peak is often referred to as the bridge region and arises from the continuous dissociation of antigen from the complex during the non-equilibrium separation^30–35^. As the antigen dissociates, its higher mobility opposes the EOF, which eventually sweeps it past the detection zone. Finally the additional peaks are observed in Fig. 3B, C, which are also present in control serum samples, are assigned to sample autofluorescence. As shown later, the most prominent peak near 79 s aligns with the albumin peak in the SPE.

**Figure 4:**
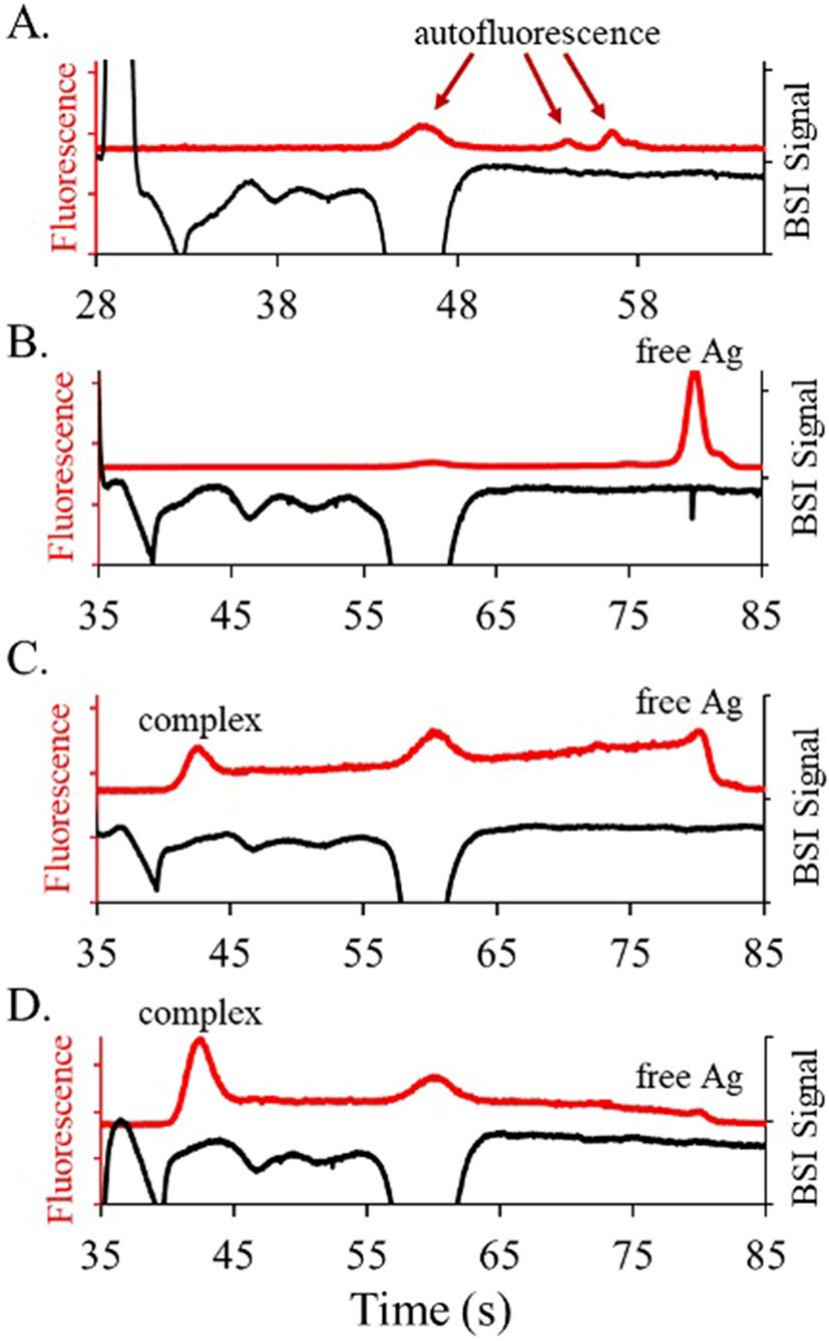
(**A**) Simultaneous SPE (BSI signal inverted for clarity) and fluorescence electropherograms for serum samples (7 mg/mL) separated in 20 mM CHES at pH 10 (500 V/cm). Peaks observed in the fluorescence signal arise from autofluorescence from the serum samples. Panels B through D show serum samples spiked with 38 nM fluorescein and incubated with (**B**) 0 nM, (**C**) 200 nM, and (**D**) 334 nM of the anti-fluorescein antibody. With no antibody present, the fluorescence electropherogram contains one peak for the free antigen. As the concentration of antibody increases, a peak associated with the complex increases and the free antigen peak decreases. The bridging region connecting the two peaks arises from the continual dissociation of complex during the non-equilibrium separation process, as discussed previously. The first peak following the EOF in the BSI electropherogram is due to a system peak.

To illustrate the dual SPE and immunoassay capabilities, Fig. 4 shows the simultaneously measured BSI and fluorescence electropherograms for three different antibody levels. For these experiments, the fluorescein antigen was added to the 7 mg/mL serum samples to a final concentration of 38 nM. The serum samples were then incubated with 0 nM, 200 nM, and 334 nM of the anti-fluorescein antibody and separated to yield the electropherograms shown in Fig. 4A, B, C, respectively.

The fluorescence electropherograms in Fig. 4 show that increasing levels of the antibody leads to increasing levels of the bound complex and decreasing levels of the free antigen as expected. As is most easily seen in Fig. 4B, there is a bridging region between the bound and unbound peaks due to the dissociation of the bound complex during the separation process. This leads to the continuous generation of free antigens during the separation as previously discussed. The presence of bound and unbound species, along with the bridge region shows that the separation time is competitive with the equilibrium kinetics. Although beyond the scope of the current study, previous studies have shown that kinetic parameters can be extracted from the bridge feature^35^. Comparison of both signals in Fig. 4 confirms that the immunocomplex migrates with the gamma globulin region in the BSI signal as expected. A comparison of both electropherograms also confirms that the autofluorescence peak observed near 60 s, corresponds with the large albumin peak seen in the BSI signal, as previously discussed.

**Figure 5:**
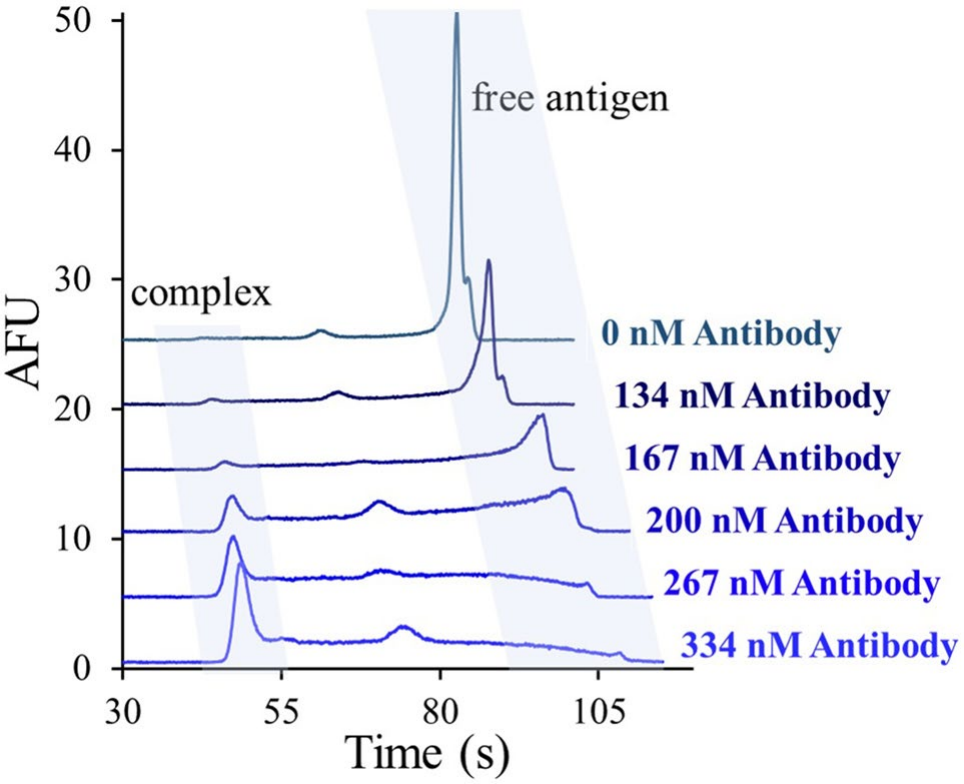
Fluorescence electropherograms for serum samples (7 mg/mL) spiked with 38 nM fluorescein and incubated with the indicated concentration of anti-fluorescein antibody. As before, the separation was carried out in 20 mM CHES at pH 10, using a field strength of 500 V/cm. The shift in the electropherograms arises from drift in the EOF.

To better characterize antigen binding, Fig. 5 shows a series of fluorescence electropherograms measured as a function of antibody level. As before, 7 mg/mL serum samples spiked with 38 nM fluorescein antigen were pre-incubated with the indicated levels of antibody. The electropherograms reveal an increase in the immunocomplex peak and decrease in free antigen peak with increasing antibody concentrations.

**Figure 6:**
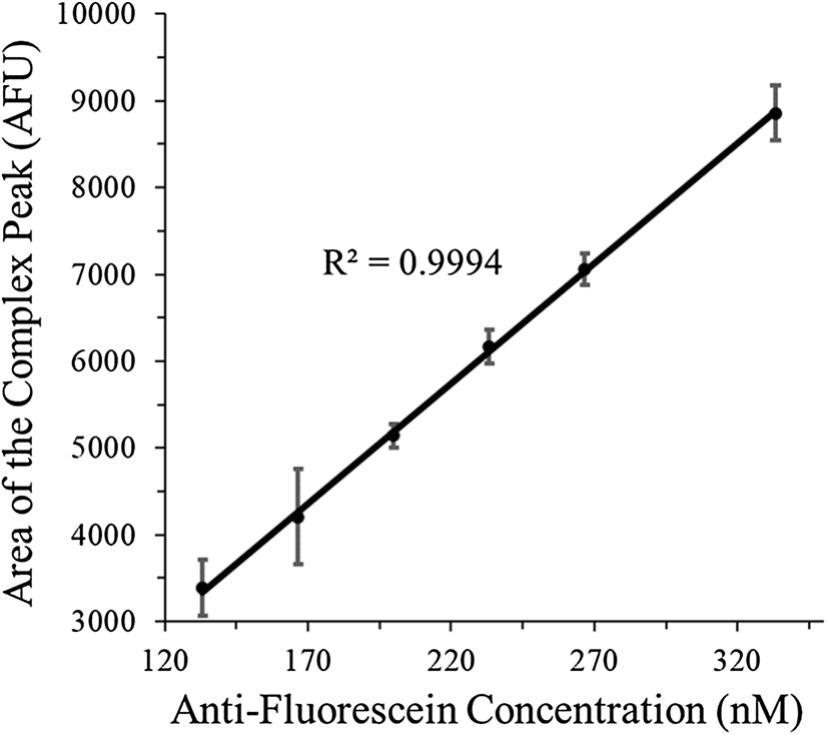
Calibration plot constructed using the electropherograms shown in Fig. 5. Peak area of the complex peak was calculated following the procedure described in ref^34^.

Integrating the areas under the peaks due to the bound complex leads to the calibration plot shown in Fig. 6. Peak areas were calculated following previous reports, resulting in excellent linearity over the limited antibody range studied^34^. From the calibration plot in Fig. 6, a 23 nM limit of detection (LOD) and 70 nM limit of quantification (LOQ) were calculated using the standard deviation of the response divided by the slope, multiplied by 3.3 and 10, respectively. For larger antibody ranges, the resulting non-linear curves can still provide quantitative results as previously reported^27,40,41,45,59^.

**Figure 7:**
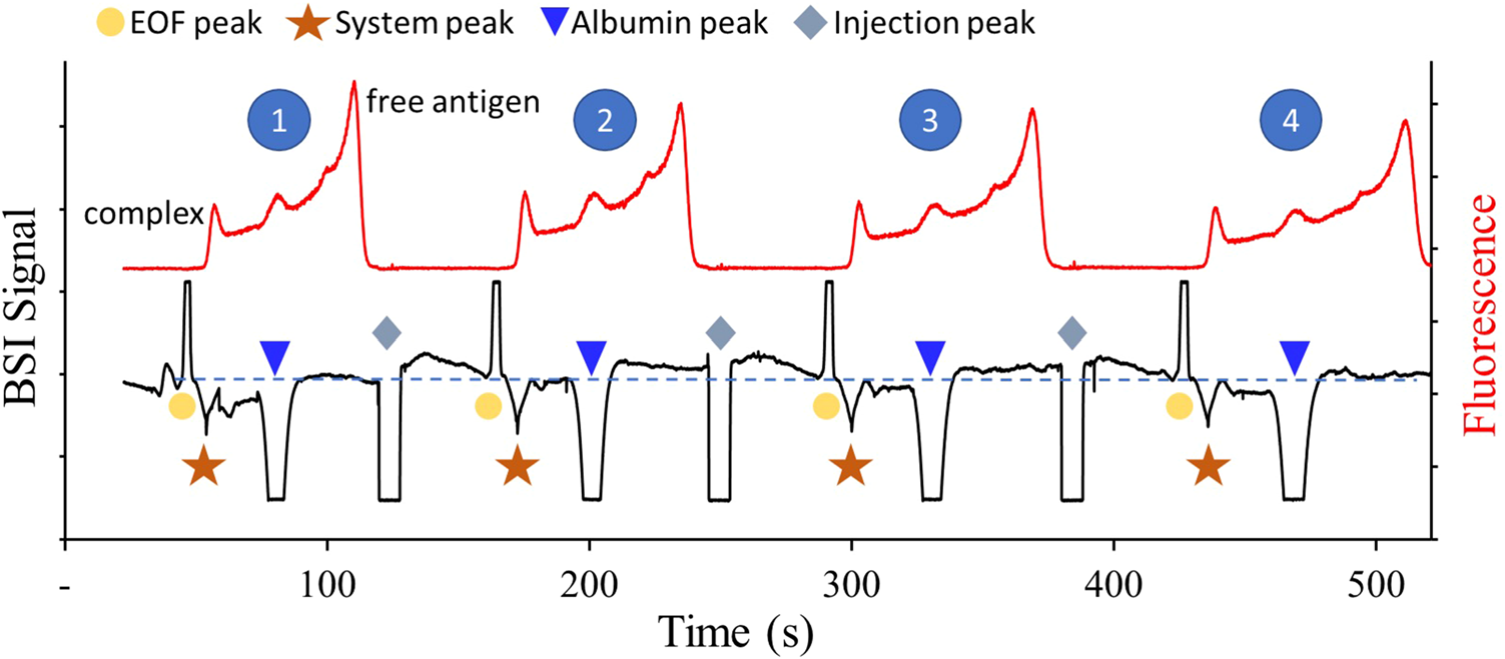
Repeated injections of a 7 mg/mL serum sample incubated with 38 nM fluorescein and 200 nM anti-fluorescein antibody. The separation was carried out in 20 mM CHES at pH 10 at a field strength of 500 V/cm.

Protein separation with CZE is often complicated by protein adsorption to the capillary walls, leading to band broadening, low separation efficiency, and poor repeatability^64–66^. This is often mitigated through the use of dynamic coatings added to the buffer^67,68^, chemical modification of the capillary surface^63,69,70^, or screening the interactions using high ionic strength buffers at elevated pH. For the HSCE approach, the pH of the buffer, along with the reduced capillary length and rapid migration through the capillary all contribute to lower protein adsorption at the capillary walls^71^. This is illustrated by the repeated injection and separation shown in Fig. 7, for a serum sample pre-incubated with 38 nM fluorescein and 200 nM of the anti-fluorescein antibody. While there are indications for reversible protein adsorption, the return of both signals to baseline levels and small variation in EOF (RSD = 2.26%) suggests that irreversible protein absorption is minimal^72,73^.

**Figure 8:**
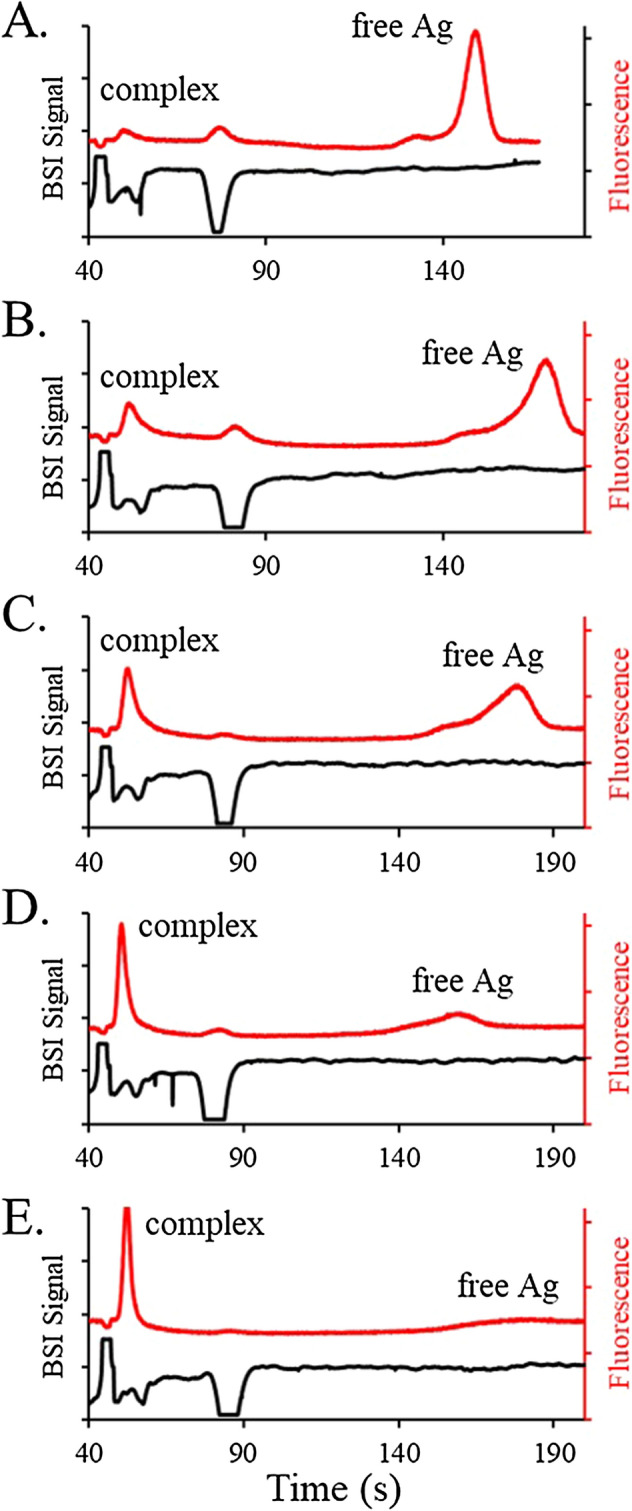
Simultaneous SPE (BSI signal inverted for clarity) and fluorescence electropherograms for serum samples (7 mg/mL) spiked with fluorescein and anti-fluorescein, separated in 50 mM barbital buffer at pH 8 (500 V/cm). The serum samples were spiked with 38 nM fluorescein and incubated with (**A**) 134 nM, (**B**) 167 nM, (**C**) 200 nM, (**D**) 267 nM, and (**E**) 334 nM of the anti-fluorescein antibody.

The elevated pH used to ensure that all the proteins are charged can also degrade immunoassays by reducing the antigen–antibody affinity. To explore the magnitude of this effect, preliminary HSCE separations in barbital BGE at pH 8 were conducted. Barbital is rarely used in CZE given its absorption in the UV, but it is compatible with the BSI signal used here. Figure 8 shows the simultaneously measured BSI and fluorescence electropherograms for serum incubated with fluorescein and the anti-fluorescein antibody. The SPE profile measured in the BSI signal shows a large peak due to albumin and a smaller peak due to transferrin. The other protein bands are not well-resolved making this particular BGE formulation unsuitable for SPE. The immunoassay measured in the fluorescence electropherogram, however, shows well separated peaks for the free antigen and complex. The lack of a bridging region between the two peaks suggest that the complex is more stable at the lower pH, with little dissociation taking place on the timescale of the separation. This improves immunoassay quantification and may provide better dual diagnostics, once conditions are found that optimize the SPE.

The combination of completely universal BSI detection with a highly specific fluorescence signal provides two complimentary measures of sample properties, each with diagnostic value. The rapid analysis times of HSCE improves the throughput for clinical applications and enables immunoassay measurements of antigen–antibody complexes before significant dissociation takes place. Conditions are shown that provide characteristic SPE profiles and enable immunoassay quantification, simultaneously. At high pH, where the antigen–antibody affinity is reduced and the complex is less stable, the fluorescence electropherogram exhibits a significant bridge region connecting the complex and free antigen peaks in the electropherogram. This region arises from the continuous dissociation of antigen from the complex and is essentially eliminated at lower pH, where the complex is more stable. Measurements done at pH 8 reveal fluorescence electropherograms with well-defined, baseline separated peaks for the complex and free antigen. This potentially improves immunoassay performance but degrades the SPE separation where the characteristic profile is not recovered in the BSI electropherogram. Further exploration of separation conditions best suited for both SPE and immunoassays, will potentially lead to new, rapid methods for diagnostics using dual detection HSCE.

## Conclusions

Refractive index detection using BSI is combined with fluorescence measurements to develop an HSCE platform capable of simultaneous SPE and immunoassay quantification. The HSCE platform uses an ultra-thin wall separation capillary to reduce Joule heating and a short length to promote rapid separation. SPE profiles in boric acid, arginine, and CHES buffers were measured with BSI and showed the characteristic five major protein bands expected for SPE. For the immunoassay, fluorescein and its antifluorescein monoclonal antibody were used as the test system. For the buffers found appropriate for SPE, only CHES enabled sufficient quantification of the immunocomplex. With fluorescein held constant in the serum sample, incubation with increasing levels of antibody resulted in growth of the immunocomplex peak and associated decreases in the free antigen peak in the fluorescence electropherograms. Comparison with the simultaneous SPE shows that the immunocomplex migrates in the gamma region as expected and calibration plots resulted in a LOD of 23 nM and LOQ of 70 nM for these preliminary studies. Both the BSI and fluorescence signals return to baseline levels after repeated sample injections, suggesting protein adsorption to the capillary walls is minimal. Finally, preliminary immunoassays carried out in a buffer at pH 8 leads to increased stability of the immunocomplex and improved separation of the complex and free antigen. While further experiments are needed to improve the SPE separation, these conditions lead to fluorescence electropherograms that lack the bridge region and are easily quantified.

## Materials and methods

### Chemicals

Analytical grade N-cyclohexyl-2-aminoethanesulfonic acid (CHES), L-arginine, boric acid, sodium hydroxide, barbital buffer and fluorescein were obtained from Sigma Aldrich (St. Louis, Missouri). Mouse anti-fluorescein monoclonal antibody was obtained from Millipore Corporation (Temecula, CA) and lyophilized powder of human serum was purchased from Thermo Fisher (Fair Lawn, New Jersey). All the chemicals were used without further purification.

Boric acid buffer (100 mM, pH 10), arginine buffer (100 mM, pH 11), CHES buffer (20 mM, pH 10) and barbital buffer (50 mM pH 8) were prepared in ultrapure water and their pH adjusted using 1 M sodium hydroxide solution. All the solutions were stored at 4 °C and allowed to equilibrate at room temperature before use. A stock solution of serum was prepared in ultrapure water at a concentration of 20 mg/mL and diluted to the appropriate final concentration. Sample mixtures were prepared by combining appropriate volumes of stock solutions of serum, fluorescein, and the anti-fluorescein antibody together and diluting to the desired concentrations using ultra-pure water.

### Experimental apparatus

The custom-designed HSCE is similar to that recently described^74^. The separation takes place in a 10 cm long total length fused silica capillary (50 μm i.d., 80 μm o.d.; VitroCom, Mountain Lakes, NJ) with a 8 cm length-to-detector. The capillary is mounted flush on the top of a copper baseplate which has a thermoelectric Peltier cooler (TEC1-12710) mounted to the underside. With the exception of small regions around the detection zone and ends of the capillary, the length of the capillary is covered with thermal paste to make good thermal contact with the underlying copper baseplate. A temperature controller (Stanford Research Systems LDC 501, Sunnyvale, CA) monitors the baseplate temperature and adjusts the Peltier cooler to maintain the temperature at the desired value with ± 0.004 °C.

An o-ring enclosure seals the outlet of the capillary for applying an under pressure. This is used for conditioning the capillary and hydrodynamic sample injections. The inlet side of the capillary overhangs the copper baseplate and extends into the meniscus of solutions held in open centrifuge tubes. The tubes are held in a computer controlled rotary stage that can quickly position the appropriate solution at the capillary inlet to exchange solutions or load samples into the capillary. Platinum electrodes for the separation voltage are positioned near the inlet and outlets of the capillary and extend into the solution resevoirs.

The HSCE is supported on a Zeiss Axiovert 100 TV inverted fluorescence microscope. For the BSI signal, the 488 nm line from an argon-krypton laser (Coherent, Innova 70, Santa Clara, CA) is focused into the detection zone of the capillary. The back-scattered interference pattern is reflected towards a split photodiode using a beam splitter. Two quadrants of the photodiode are aligned on the fringe pattern, and the differential signal is amplified and recorded as the BSI signal. The same 488 nm laser line also excites fluorescence in the capillary, which is collected from below using a 0.75 NA, 80 × ultralong working distance objective (Olympus ULWD NeoSPlan 80). The collected light is filtered (Chroma 500 nm long pass) to remove residual excitation light and the fluorescence is detected on a single-photon avalanche diode (SPAD, EG&G). The digital output of the SPAD is sent to a counter (National Instruments, USB-6251) and binned in software written in LabVIEW.

Before each separation, the capillary is conditioned for 5 min with 0.1 M NaOH, using an under pressure applied at the capillary outlet to flow the solution. This is followed with 5 min treatments with ultra-pure water and 5 min with the BGE. For all separations reported here, samples are pressure injected for a duration of 150 ms at 65 kPa. Following injection, all samples are separated in normal polarity mode using field strengths ranging from 500 to 700 V/cm (Spellman, CZE 1000R).

## Data Availability

The datasets generated during and/or analysed during the current study are available from the corresponding author on reasonable request.
